# Late diagnosis of attention deficit hyperactivity disorder and cocaine abuse

**DOI:** 10.1192/j.eurpsy.2023.735

**Published:** 2023-07-19

**Authors:** C. De Andrés Lobo, C. Vallecillo Adame, T. Jiménez Aparicio, M. Queipo de Llano de la Viuda, G. Guerra Valera, A. A. Gonzaga Ramírez, M. Fernández Lozano, N. Navarro Barriga, M. J. Mateos Sexmero, B. Rodríguez Rodríguez, M. Calvo Valcárcel, M. Andreo Vidal, M. P. Pando Fernández, P. Martínez Gimeno, I. D. L. M. Santos Carrasco, J. I. Gonçalves Cerejeira, A. Rodríguez Campos

**Affiliations:** 1 Psychiatry; 2 Hospital Clínico Universitario de Valladolid, Valladolid; 3 Psychiatry, Hospital Universitario Infanta Cristina, Madrid; 4 Psychiatry, Hospital Río Carrión, Palencia, Spain

## Abstract

**Introduction:**

Adult ADHD diagnosis sometimes represents a challenge for the clinician, due to the comorbid psychiatric diseases that are often associated and which complicate de recognition of the primary symptoms of ADHD. The prevalence of ADHD in adult populations is 2’5% and it is a relevant cause of functional impairment.

**Objectives:**

Presentation of a clinical case of a male cocaine user diagnosed with adult ADHD.

**Methods:**

Literature review on adult ADHD and comorbid substance abuse.

**Results:**

A 43-year-old male who consulted in the Emergency Department due to auditory hallucinosis in the context of an increase in his daily cocaine use. There were not delusional symptoms associated and judgment of reality was preserved. Treatment with olanzapine was started and the patient was referred for consultation. In psychiatry consultations, he did not refer sensory-perceptual alterations anymore, nor appeared any signals to suspect so, and he was willing to abandon cocaine use after a few appointments. He expressed some work concerns, highlighting that in recent months, in the context of a greater workload, he had been given several traffic tickets for “distractions.” His wife explained that he had always been a inattentive person (he forgets important dates or appointments) and impulsive, sometimes interrupting conversations. In the Barkley Adult ADHD Rating Scale he scored 32 points.

He was diagnosed with adult ADHD and treatment with extended-release methylphenidate was started with good tolerance and evolution, with improvement in adaptation to his job and social environment. Since then, the patient has moderately reduced the consumption of drugs, although he continues to use cocaine very sporadically.

**Conclusions:**

Early detection of ADHD and its comorbidities has the potential to change the course of the disorder and the morbidity that will occur later in adults. Comorbidity in adult ADHD is rather the norm than the exception, and it renders diagnosis more difficult. The most frequent comorbidities are usually mood disorders, substance use disorders, and personality disorders. Treatment of adult ADHD consists mainly of pharmacotherapy supported by behavioral interventions. When ADHD coexists with another disorder, the one that most compromises functionality will be treated first and they can be treated simultaneously. The individual characteristics of each patient must be taken into account to choose the optimal treatment.

**Disclosure of Interest:**

None Declared

